# Successful Rescue of Ventricular Fibrillation Electrical Storm Secondary to Acute Myocardial Infarction in a Patient Presenting to a District General Hospital: A Case Report

**DOI:** 10.7759/cureus.73959

**Published:** 2024-11-18

**Authors:** Jhiamluka Solano, Gedoni Eni, Aishwarya Viswanath, Basem Enany

**Affiliations:** 1 Education, Academy of Medical Educators, Cardiff, GBR; 2 Cardiology, Scunthorpe General Hospital, Scunthorpe, GBR; 3 Internal Medicine, Scunthorpe General Hospital, Scunthorpe, GBR; 4 Interventional Cardiology, Hull University Teaching Hospital National Health Service Trust, Hull, GBR

**Keywords:** cardiac electrical storm, human factors in debriefing, lucas device, out-of-hospital cardiac arrest, st-elevation myocardial infarction (stemi)

## Abstract

Ventricular arrhythmia is a critical and challenging cardiovascular complication of myocardial infarction (MI). An electrical storm (ES), characterised by three or more episodes of sustained ventricular arrhythmia within 24 hours, poses a significant life-threatening risk. Standard management includes advanced life support (ALS) protocols and specialised pharmacological interventions. We present the case of a 43-year-old female who presented to the emergency department (ED) following an out-of-hospital ventricular fibrillation (OOHVF) arrest, with the return of spontaneous circulation (ROSC) achieved after multiple defibrillation shocks. Electrocardiography (ECG) revealed anterior ST-segment elevation MI (STEMI) involving the left anterior descending (LAD) artery. During her ED stay, she experienced recurrent ventricular fibrillation (VF) arrests requiring repeated defibrillation, adrenaline, amiodarone, and thrombolysis with alteplase. She was subsequently intubated and transferred to a primary percutaneous coronary intervention (PPCI) centre with intensive care support. Angiography confirmed a 100% occlusion of the LAD, which was successfully treated with stenting. The patient was admitted to the intensive care unit (ICU) and later discharged with full neurological recovery, on secondary prevention and heart failure therapy, with follow-up planned. This case underscores the complexity of managing electrical storms in MI, particularly in non-PPCI centres. It emphasises the importance of thrombolysis as an early reperfusion strategy in STEMI, especially when PPCI is not immediately available.

## Introduction

Ventricular arrhythmias represent a challenging and critical cardiovascular complication of myocardial infarction (MI). An electrical storm (ES) is considered a life-threatening condition characterized by multiple episodes of sustained arrhythmia, such as ventricular tachycardia or fibrillation, defined as three or more episodes of sustained arrhythmia occurring within 24 hours, at least five minutes apart, and requiring intervention due to the risk of sudden cardiac arrest if not rapidly controlled [1]. Although arrhythmias after MI are a well-known complication, the incidence has considerably decreased due to access to primary percutaneous interventions (PPCI). It is estimated that about 6% of patients with MI will develop ventricular fibrillation (VF) in the first 48 hours of admission [2]. Management is usually guided by advanced life support (ALS) algorithms and specialised pharmacological agents outside the standard ALS protocols. According to the European Society of Cardiology, ventricular arrhythmias due to underlying coronary artery disease require consideration of specific pharmacologic therapies, including amiodarone, beta-blockers, lidocaine, and mexiletine in addition to emergency revascularization in the context of acute MI [3]. Similarly, the American Heart Association (AHA) recommends starting with amiodarone and lidocaine, followed by beta-blockers in refractory cases [4]. This case report illustrates our successful management of an MI-induced ES in a patient presenting to a non-PPCI centre, highlighting the importance of ALS protocols and multidisciplinary collaboration.

## Case presentation

**Figure 1:**
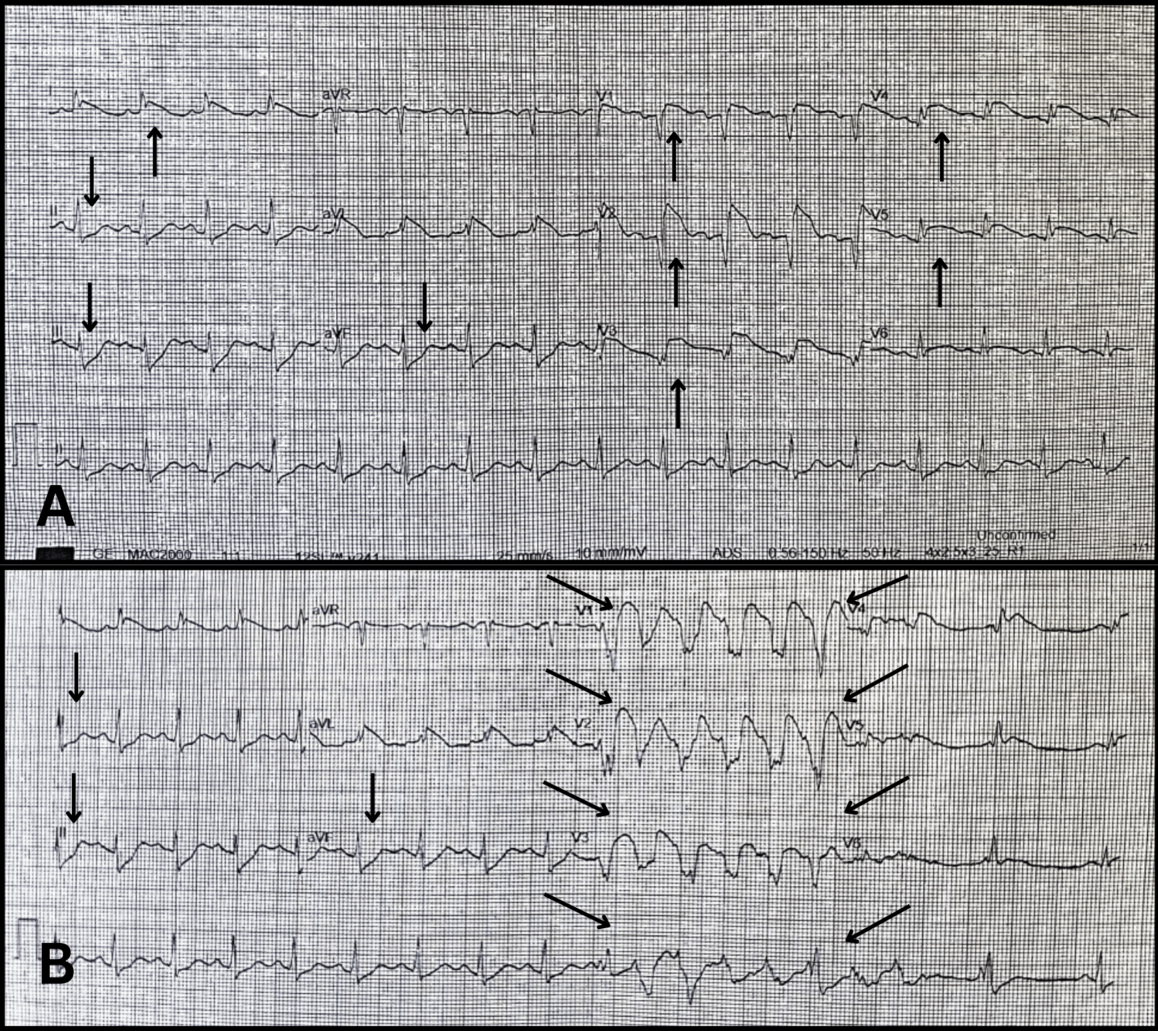
ECG obtained after the return of spontaneous circulation (ROSC) A. ECG showing ST-segment elevation in the anterolateral leads (leads V1 to V4) with reciprocal ST-segment depression in the inferior leads (leads II, III, and aVF). B. ECG showing ST-segment depression in the inferior leads (leads II, III, aVF) and runs of non-sustained ventricular tachycardia (VT) (leads V1 to V3).

In January 2024, a 43-year-old female with a medical history of elevated body mass index (BMI), ovarian cyst, asthma, and tobacco use presented to the emergency department (ED) after the return of spontaneous circulation (ROSC) following a VF arrest. Her symptoms before the cardiac arrest included chest pain radiating to her left arm and jaw, associated with nausea and vomiting. During transit to the ED, she suffered multiple VF arrests requiring over ten defibrillation shocks. Upon arrival in the resuscitation bay, the patient experienced another VF episode, necessitating further defibrillation, which successfully restored circulation. Following the ROSC, the patient was noted to be agitated and wheezy. An electrocardiogram (ECG) revealed an anterolateral ST-elevation myocardial infarction (STEMI) (Figures 1A, 1B). She continued to have recurrent VF arrests with varying intervals, at least 10 minutes apart, requiring repeated defibrillation. She was intubated and mechanically ventilated, and her initial blood test revealed normal potassium, severe acidosis with a pH of 6.7 (7.32-7.43), a troponin T of 1,415 ng/L (0-10 ng/L), and a markedly elevated lactate level of 19 mmol/L (0.5-2.2 mmol/L).

Resuscitative measures included the administration of amiodarone, adrenaline, and thrombolysis with alteplase. Cardiopulmonary resuscitation (CPR) was prolonged, utilising a LUCAS device (Jolife AB, Lund, Sweden) for mechanical chest compressions, with a cumulative time of supported circulation of one hour. Initial contact with the specialist centre prompted plans for a CT head scan, but the scan was abandoned after the patient experienced another VF arrest while in the scanner. Further resuscitative efforts, including a second dose of thrombolysis, multiple defibrillation shocks, 1.2 g of amiodarone and several doses of adrenaline, were administered. Although lidocaine was prepared, it was not administered.

**Figure 2:**
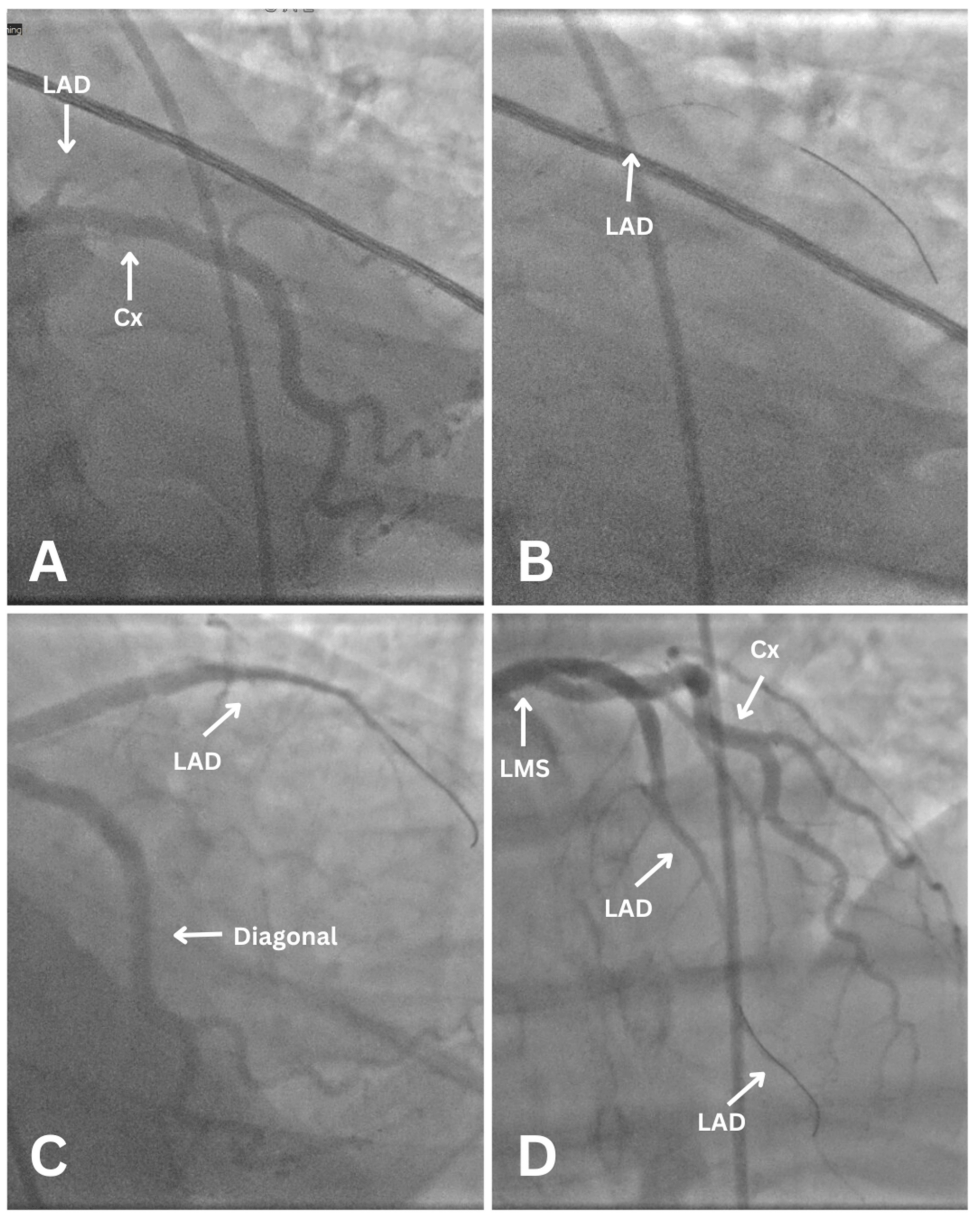
Angiogram views during primary percutaneous coronary intervention (PPCI) PPCI: (A) Left anterior oblique (LAO) caudal showing proximal left anterior descending (LAD) coronary artery total occlusion. (B) Right anterior oblique (RAO) caudal showing run-through wire and ballooning to LAD (arrow). (C) RAO caudal is showing post-LAD stent. (D) LAO cranial view is showing Thrombolysis in Myocardial Infarction (TIMI) III flow in LAD post-stenting.

After further consultation with cardiology, she was transferred to a nearby catheterisation laboratory with intensive care support for rescue percutaneous coronary intervention (PCI). A pre-PPCI emergency bedside echocardiogram revealed severely impaired left ventricular systolic function, with regional wall motion abnormalities consistent with ischemia in the left anterior descending (LAD) artery territory. Unfortunately, the images were not stored, and only the report was available for reference. Coronary angiography, performed via the right femoral artery, identified a complete occlusion of the proximal LAD, which was successfully treated with stenting (Figure 2).

After an unremarkable CT head scan, she was commenced on tirofiban infusion and transferred to the intensive care unit (ICU) for post-ROSC care. After a week in the ICU, the patient demonstrated full neurological recovery and was transferred to the medical ward. She was discharged on dual antiplatelet therapy, along with secondary prevention and heart failure medications. A follow-up angiogram was planned to evaluate the right coronary artery further, and future out-patient follow-up was arranged. She tolerated the prescribed medications well, with stable vital signs at discharge. On follow-up, she had a full recovery and tolerated the indicated heart failure therapies. She continued to have routine follow-ups with the heart failure nurses and cardiologists. 

## Discussion

Electrical storm (ES) typically occurs in patients with underlying structural heart diseases, such as ischemic heart disease, cardiomyopathies, or channelopathies [5]. It is a rare but life-threatening complication of acute myocardial infarction (AMI), most associated with lesions of the LAD or right coronary artery. The pathophysiology of ES is complex, requiring a combination of predisposing factors, including structural or electrical heart disease, an external triggering event, and autonomic dysregulation, typically characterised by sympathetic hyperactivity [6,7].

ES is most often observed in older male patients with structural heart disease, reduced ejection fraction, advanced heart failure, and multiple cardiovascular comorbidities [7,8]. However, in this case, the patient was a younger female with risk factors including smoking and obesity but no prior diagnosis of coronary artery disease (CAD). Emerging evidence on ischemic cardiomyopathies in previously asymptomatic individuals [9,10] suggests the need to lower the threshold for investigating patients in this category to reduce morbidity and mortality. While the role of sympathetic dysregulation in the genesis of ES is well-established, the exact mechanisms remain unclear. Animal and human studies suggest excessive sympathetic nerve sprouting and cardiac hyperinnervation as potential contributors to post-MI arrhythmogenicity [11,12]. Further research into the specific pathways of arrhythmogenesis post-MI is essential to improve management for patients presenting with ES as the initial manifestation of AMI.

The therapeutic approach to ES is primarily determined by local expertise and available resources [5]. According to the 2022 European Society of Cardiology (ESC) guidelines [13], ventricular arrhythmias (VA) before reperfusion are more common than those occurring during or after reperfusion. Early revascularisation is the optimal strategy to prevent VAs in the setting of acute STEMI, and beta-blockers are recommended before PCI if not contraindicated [13].

In non-PPCI centres, patients are typically stabilised and transferred for emergency PCI. In this case, the patient was unstable, necessitating thrombolytic therapy with intravenous alteplase for attempted revascularisation, administered twice during episodes of VF arrest, with a total dose of 100 mg. A Chinese study [14] on the two-year mortality of fibrinolytic-treated STEMI patients found that successful fibrinolysis, particularly with alteplase, and rescue PCI were predictors of survival. They also noted that 30-day mortality comprised a substantial portion of overall mortality at two years, with only about 20% of patients undergoing rescue PCI after failed fibrinolysis, which would likely have improved survival. Similarly, an Irish study [15] reported a 71% successful revascularisation rate (Thrombolysis in Myocardial Infarction (TIMI) II-III flow) in STEMI patients treated with fibrinolysis, compared to 29% in cases of failed revascularisation. However, the fibrinolytic agent was not specified. These findings demonstrate that fibrinolysis is inferior to PCI (as was the case in our patient), and it remains a valuable treatment option, particularly in unstable patients at non-PCI centres. As witnessed during the COVID-19 pandemic, where the use of fibrinolysis increased due to procedural delays [16], clinicians in district general hospitals and non-cardiac specialist centres must be well-versed in thrombolysis protocols.

To manage ES with recurrent ventricular tachyarrhythmias, the 2022 ESC guidelines [13] recommend addressing elevated sympathetic tone, preferably using non-selective beta-blockers such as propranolol or sedation and amiodarone therapy. Other antiarrhythmic agents, such as procainamide or lidocaine, are reserved for specific situations. Mechanical ventilation and autonomic modulation, including percutaneous stellate ganglion blockade and left cardiac sympathetic denervation, are recommended for selected patients with refractory VAs [13]. In non-cardiac referral centres, the therapeutic options are often limited to defibrillation, pharmacotherapy, and sedation with mechanical ventilation.

The repetitive nature of CPR in ES can be physically challenging, especially in smaller hospitals with limited staff. Although mechanical chest compression devices such as LUCAS have received mixed reviews, with a meta-analysis [17] reporting inconclusive results, other studies have suggested improved rates of ROSC [18], especially in prolonged resuscitation. In this case, using the LUCAS device was instrumental in mitigating the physical burden on the team. Moreover, this case illustrates that total downtime and pH values during arrest are not always definitive predictors of poor outcomes.

Challenges and learning points

Managing this case in a district general hospital without PPCI services presented several challenges, including decisions about balancing patient stabilisation against the risks of transferring for PPCI and logistical delays in arranging emergency transport. Miscommunication regarding the possibility of a head injury at the initial VF arrest led to an unnecessary CT head scan request, further delaying the transfer. This case reiterates the importance of vigilant monitoring post-ROSC and the flexible application of ALS protocols in managing complex arrhythmias. It emphasises the need for front-door clinicians to remain familiar with the guidelines for managing ventricular tachycardia (VT)/VF storms and the crucial role of multidisciplinary collaboration in such cases. Clear, accurate communication between emergency and specialist teams is critical to minimising delays in definitive treatment and optimising outcomes.

## Conclusions

This case illustrates the complexity of managing ESs in MI, particularly in non-PPCI centres. It highlights the significant burden of intensive resuscitation on small cardiac arrest teams and the essential role of multidisciplinary collaboration. Additionally, the case report emphasises the importance of thrombolysis as an early reperfusion strategy in STEMI, especially when PPCI is not immediately available. Clinicians must maintain proficiency in thrombolysis administration while awaiting PCI in stabilised patients.
